# Geographic and urban–rural disparities in the total prevalence of neural tube defects and their subtypes during 2006–2008 in China: a study using the hospital-based birth defects surveillance system

**DOI:** 10.1186/1471-2458-13-161

**Published:** 2013-02-22

**Authors:** Xiaohong Li, Jun Zhu, Yanping Wang, Dezhi Mu, Li Dai, Guangxuan Zhou, Qi Li, He Wang, Mingrong Li, Juan Liang

**Affiliations:** 1 National Center for Birth Defects Monitoring, West China Second University Hospital, Sichuan University, No 17, section 3, Ren Min Nan Lu, Chengdu, Sichuan, China; 2 West China School of Public Health, Sichuan University, No 17, section 3, Ren Min Nan Lu, Chengdu, Sichuan, China; 3 National Office for Maternal and Child Health Surveillance, West China Second University Hospital, Sichuan University, No 17, section 3, Ren Min Nan Lu, Chengdu, Sichuan, China; 4 Key Laboratory of Obstetric & Gynecologic and Pediatric Diseases and Birth Defects of Ministry of Education, Sichuan University, No 17, section 3, Ren Min Nan Lu, Chengdu, Sichuan, China; 5 Prenatal Diagnosis Department, West China Second University Hospital, Sichuan University, No 17, section 3, Ren Min Nan Lu, Chengdu, Sichuan, China

**Keywords:** Neural tube defects, Prevalence ratio, Geographic disparity, Urban–rural disparity

## Abstract

**Background:**

Previous reports on the prevalence of neural tube defects (NTDs) in China did not include cases of NTDs that were less than 28 weeks of gestational age (GA) and hence did not accurately reflect the total prevalence of NTDs or the geographic and urban–rural disparities in their prevalence. This article includes cases of NTDs that were less than 28 weeks of GA.

**Methods:**

Data used in this study were collected from 2006 to 2008 using a nationwide hospital-based registry, the Chinese Birth Defects Monitoring Network. The total prevalence ratio (PR) of NTDs and their subtypes, the ratios of PR (PRR), and 95% confidence intervals (CI) were used to analyse geographic disparities at both the regional (north, south) and provincial levels and to analyse disparities between rural and urban areas.

**Results:**

Overall, the total PR of NTDs was 14.0 per 10,000 births. The PRR of NTDs of rural women between the north and south region was 2.26 (95% CI: 2.04-2.52), which was much higher than that of urban women (PRR: 1.56, 95% CI: 1.41-1.72). The three subtypes of NTDs had different geographic distribution at the level of province. The urban–rural PRR of NTDs was 2.14 (95% CI: 1.94-2.34) in the north but only 1.47 (95% CI: 1.31-1.66) in the south.

**Conclusions:**

There is a high total prevalence of NTDs, which remains one of the major public health concerns in China. Eliminating the geographic and urban–rural disparities in the disease burden is a priority for future intervention.

## Background

Neural tube defects (NTDs) are one of the most common birth defects. These defects are caused by abnormal development of the neural tube during embryonic life, which produces injuries of the central nervous system (the brain and spinal cord). In the past several decades, NTDs have been the object of more intense epidemiological studies than other types of birth defects because of the more severe outcomes of NTDs, including fatalities or disability. Although the causes of NTDs are not completely understood, many risk factors, such as genetics, the environment, and nutritional factors have been identified [1-4]. Several effective measures for NTD prevention and management have been developed [5,6]. A previous published report indicated that China has one of the highest prevalence of NTDs in the world [7]. In the last two decades, many epidemiological studies of NTDs in China have focused on the large residential (urban–rural) and geographic (northern-southern) disparities in NTDs [7-10]. However, most of these studies only examined cases of NTDs that were greater than or equal to 28 weeks of gestational age (GA), and terminations of pregnancy or births (live and still) affected with NTDs that were less than 28 weeks of GA were not included in their analysis. The official PR of NTDs in China,which only included the NTDs affecting individuals that were greater than or equal to 28 weeks of GA, has declined to 7.18 per 10,000 births in 2008 from 27.38 per 10,000 births in 1987 [7,11]. The decline has moved NTDs to be ranked fourth most prevalent among all congenital malformations. Because of its high sensitivity, prenatal ultrasound is widely used to diagnosis NTDs. A large proportion of pregnancies with NTDs can be diagnosed and terminated before 28 weeks of GA [12,13]. Cases of NTDs in pregnancies that are greater than or equal to 28 weeks of GA only represents 53% of the total cases. [14] This means that the previously reported prevalence of NTDs does not entirely capture the true prevalence of NTDs in China. In addition, we were unable to identify whether the residential and geographic disparities in the PR of NTDs were mainly caused by variation in the quality of prenatal diagnosis. Although it is impossible to accurately calculate the incidence of NTDs of a country or region/area [15,16], we should not ignore cases of NTDs that are less than 28 weeks of GA. Several congenital anomalies in the registry system of developed countries can provide data on the prevalence of NTDs including terminations of pregnancy for fetal anomaly (TOPFA) regardless of GA [17]. Studies on the total prevalence of NTDs and its epidemiological characteristics can help us understand the disease burden, make effective interventions, and evaluate primary public health measures.

This study will use a newly updated database, the Chinese Birth Defects Monitoring Network (CBDMN), to investigate the epidemiological patterns of NTDs and the presence of geographic and urban–rural disparities in the total prevalence of NTDs at any weeks of GA in China during 2006–2008. New insight will be provided into the aetiology of NTDs, prevention and management of NTDs.

## Methods

### Data source

Data used in the study were retrieved from the national birth defects surveillance database maintained by CBDMN during 2006–2008. CBDMN is a hospital-based network established in 1986. It is under the supervision of the Department of Maternal and Child Health Care and Community Health of the Ministry of Health of China. The network covers approximately 517 county-level and higher hospitals throughout 116 counties or districts in 31 provinces, cities, or autonomous regions. The areas were classified according to their geographical location and socioeconomic status. These locations included a coastal region, inner land, and remote areas. A total of 641,789, 775,333, and 840,341 births (live and still) were monitored in member hospitals in these regions in 2006, 2007, and 2008 respectively. The live births monitored by CBDMN account for 5.40% to 6.25% of total live births in China with mean proportions of 5.95% based on the national number of live births. ( http://www.moh.gov.cn/zwgkzt/ptjnj/200908/42635.shtml).

### Monitoring subjects

Prior to 2006, the subjects examined in the CBDMN were all births (live and still) or TOFPAs that were greater than or equal to 28 weeks of GA (if the GA of the births were unknown, those weighing over 1000 g were also monitored). The maximal diagnosis time for birth defects was within seven days after birth. From 2006 on, according to the revised and more comprehensive requirements of the monitoring program, cases with major congenital malformation (e.g. NTDs, congenital heart defects, acromphalus, gastroschisis, limb reduction defects, etc.) that were live births, stillbirths, and TOPFAs regardless of GA in the member hospitals, must be reported to the National Center of Birth Defects Surveillance of China.

### Data collection

A three-level (county, province, and central) surveillance network and corresponding expert groups were established to undertake data collection. The procedure for CBDMN data collection has been described elsewhere [14,18]. In the member hospitals, every neonate or stillbirth is immediately examined after birth by trained healthcare professionals to screen for birth defects. Cases of abnormalities diagnosed by prenatal diagnosis and terminated before 28 weeks of GA, were reconfirmed after termination. Experts in the surveillance group, which usually includes paediatricians, obstetricians, and ultrasound specialists, at the county-level surveillance network were requested to confirm the diagnosis in each case. When the diagnosis was not clear, the staff in charge of birth defect monitoring in the member hospital (usually the nurse) collected more details (e.g. medical records and photos of the case) to be used for re-diagnosis by a group of experts at provincial-level surveillance network. The National Center of Birth Defect Monitoring holds an expert seminar annually to diagnose unknown cases using a review of the medical records and photos. If the case cannot be clearly diagnosed, it was not included in the system. For each case, the nurse is responsible for gathering information (e.g. family socioeconomic status, demographics, clinical features, obstetric items, etc.) using individualised interviews of the mothers and medical record reviews. The standardized form, “Birth Number Register Form,” is used for collecting the number of hospital delivery births by mother’s age, residential areas, infant’s sex and pregnancy outcome on a monthly basis. The “Birth Defect Register Form” is used for collecting data on birth defects. The data are entered regularly to an online reporting system for MCH surveillance ( http://zhibao.mchscn.org) by the data entry clerks at the county level. Data were submitted quarterly to the provincial level and national level to be checked by a workgroup composed of clinicians, epidemiologists, and statisticians. When errors are identified, the form is returned and verified by the workgroup.

### Statistical standards and method*s*

The CBDMN adopted the same statistical standards for recording and reporting NTDs as the International Clearinghouse for Birth Defects Surveillance and Research (ICBDSR), a nongovernmental organization responsible for international information exchange on birth defects surveillance. NTDs include anencephaly (ICD10:Q00), spina bifida (ICD10:Q05), and encephalocele (ICD10:Q01). Anencephaly includes anencephaly with spina bifida, anencephaly with encephalocele, and anencephaly with hydrocephalus, but excludes acephalia and hydranencephaly. Spina bifida with hydrocephalus or encephalocele is counted only as spina bifida. This does not include spina bifida with anencephaly, spina bifida occulta or sacrococcygeal teratoma. Encephalocele is excluded in encephalocele with spina bifida [11].

The regions of China were divided into southern and northern areas, with the provinces north of latitude 35° belonging to the northern region and those south of latitude 35° belonging to the southern region. The northern region includes 15 provinces,autonomous regions, or municipalities (Beijing, Tianjing, Hebei, Shanxi, Neimenggu, Liaoning, Jilin, Heilongjiang, Shandong, Henan, Shaanxi, Gansu, Qinghai, Ningxia, and Xinjiang), and the southern region includes 16 provinces, autonomous regions, or municipalities (Shanghai, Jiangsu, Zhejiang, Anhui, Fujian, Jiangxi, Hubei, Hunan, Guangdong, Guangxi, Hainan, Chongqing, Sichuan, Guizhou, Yunnan, and Tibet). Residential areas were categorised into urban (cities or urbanised areas or neighborhood committee) and rural (villages or countryside) areas according to the last place the mother resided for at least 1 year.

Because the number of total births at any GA cannot be obtained from the CBDMN, the prevalence ratio (PR), instead of prevalence rate or prevalence proportion, was used to describe the occurrence of NTD cases. The total PR was expressed as the number of cases of NTDs at any GA per 10,000 births (live births and stillbirths of greater than or equal to 28 weeks of GA). The 95% confidence interval (CI) for the total PR was calculated based on a Poisson distribution [15]. A ratio of the total PR (PRR) between southern and northern areas and between urban and rural areas was used to describe the geographical disparity and residential disparity, respectively. PRR_adj_ adjusted by the year and maternal age was calculated using the Poisson regression model, in which four factors (year, region, residential area, and maternal age) and one interaction items (Region*Residential area) were entered. The Pearson chi-square test was used to compare the composition of NTD subtypes between different regions.

All statistical analyses in this study were performed using SAS 9.0 software (SAS version 9.0; SAS Institute, Carey, NC, USA). The statistical significance level for α was set at 0.05.

## Results

During 2006 to 2008, a total of 3,168 cases (of which 1495 cases were less than 28 weeks of GA and accounted for 47.2%) of NTDs were identified, which yielded a total PR of 14.0 (95% CI: 13.4-14.5) per 10,000 births. The total PR of NTDs was 18.7 and 9.7per 10,000 births in the north and south, respectively, and the total PR of NTDs was 21.9 and 10.1 per 10,000 births in the rural and urban, respectively. (See the Table 1) Cases of NTDs that were less than 28 weeks of GA accounted for 44.4% and 52.1% of total NTDs in the north and south, respectively, and 39.3% and 55.9% in the rural and urban areas, respectively.

**Table 1:** The geographical and urban–rural-specific prevalence ratio of NTDs (per 10,000 births) in China, 2006-2008

**Region**	**Residential areas**		**Anencephaly**		**Spina bifida**		**Encephalocel**		**NTDs**
		N^a^	PR^b^ (95% CI)	N^a^	PR^b^ (95% CI)	N^a^	PR^b^ (95% CI)	N^a^	PR^b^ (95% CI)
North	Rural	429	10.8 (9.8-11.9)	597	15.0 (13.8-16.3)	152	3.8 (3.2-4.5)	1178	29.7 (28.0-31.4)
	Urban	311	4.5 (4.0-5.1)	399	5.8 (5.3-6.4)	136	2.0 (1.7-2.3)	846	12.4 (11.5-13.2)
South	Rural	252	7.0 (6.2-8.0)	147	4.1 (3.5-4.8)	79	2.2 (1.8-2.7)	478	13.3 (12.214.6)
	Urban	333	4.1 (3.7-4.5)	214	2.6 (2.3-3.0)	119	1.5 (1.2-1.7)	666	8.1 (7.5-8.8)
	Rural	681	9.0 (8.4-9.7)	744	9.8 (9.2-10.6)	231	3.1 (2.7-3.5)	1656	21.9 (20.9-23.0)
	Urban	644	4.3 (4.0- 4.6)	613	4.1 (3.8-4.4)	255	1.7 (1.5-1.9)	1512	10.1 (9.6-10.6)
North		740	6.8 (6.4-7.3)	996	9.2 (8.6-9.8)	288	2.7 (2.4-3.0)	2024	18.7 (17.9-19.5)
South		585	5.0 (4.6-5.4)	361	3.1 (2.8-3.4)	198	1.7 (1.5-1.9)	1144	9.7 (9.2-10.3)

^a^The number of NTDs affected cases.

^b^PR=prevalence ratio.

**Figure 1:**
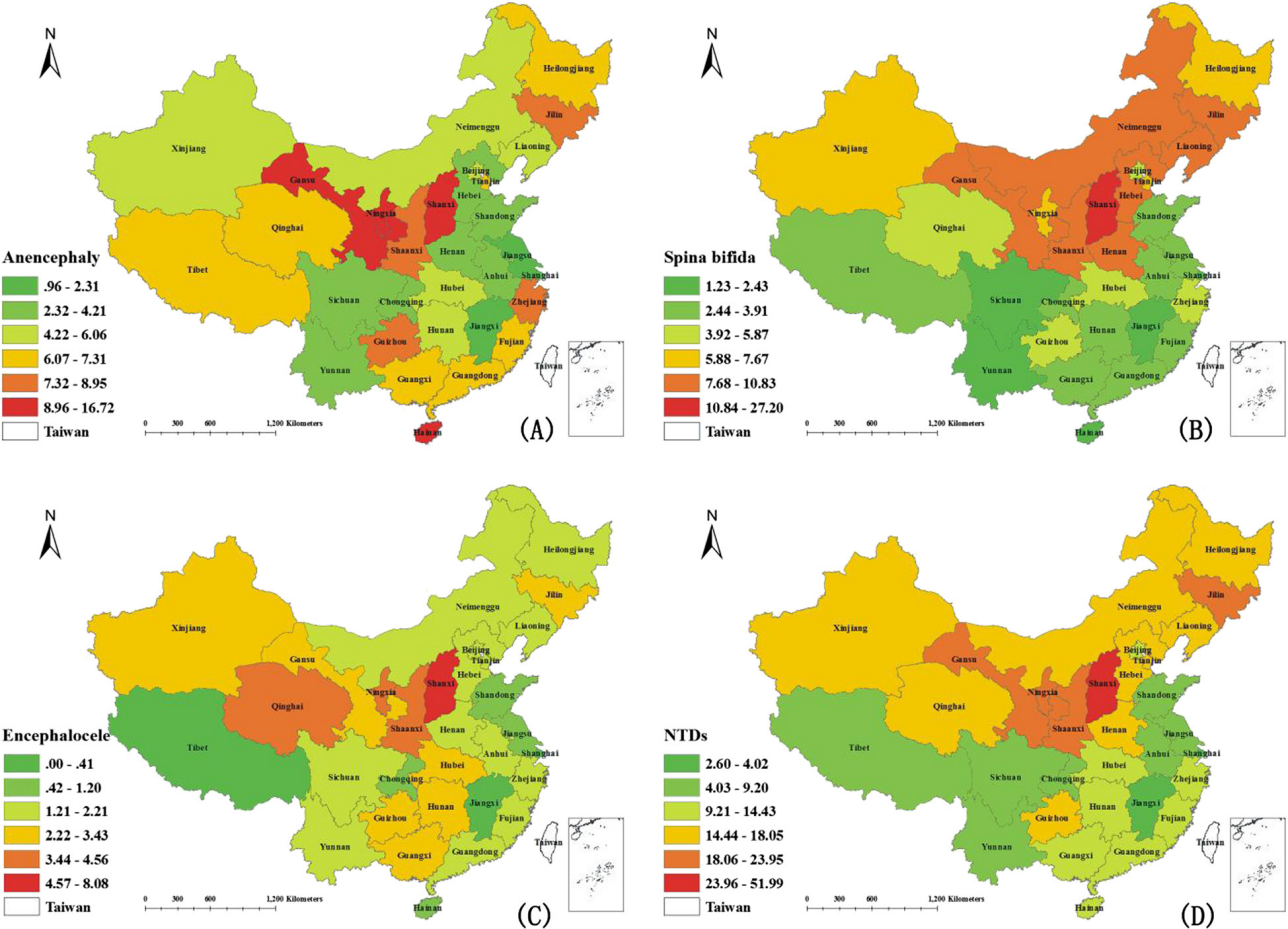
**The PR of NTDs and its subtypes in different provinces of Mainland China in 2006-2008. ** (**A**) The PR of anencephaly in different province. (**B**) The PR of spina bifida in different province. (**C**) The PR of encephalocele in different province. (**D**) The PR of NTDs in different province.

The composition of NTD subtypes also varied among the different regions (*x*^*2*^=118.80, p<0.001). In the south, anencephaly accounted for the largest proportion of NTDs cases (51.14%) while in the north, spina bifida accounted for the largest proportion (49.21%). As for the total PR of NTDs and NTD subtypes at the provincical level, the highest prevalence of NTDs was observed in the north-western provinces, such as Shanxi, Shaanxi, Ningxia, Gansu, and Jilin. The distribution of spina bifida showed a similar pattern, but anencephaly had a high prevalence in some southern provinces such as Guizhou and Hainan outside of the the north-western provinces. Encephalocele had the highest prevalence in the north-western areas. The north-eastern region of China did not have a high prevalence of encephalocele (Figure 1).

### Geographic and urban–rural disparities in the total PR of NTDs and NTD subtypes

The PRR of NTDs was 1.56 (95% CI: 1.41-1.72) in the northern-urban region compared to the southern-urban region and 2.26 (95% CI: 2.04-2.52) in the northern-rural region compared to the southern-rural region (Table 2). The total PR of spina bifida in the northern-rural region was 3.71 (95% CI: 3.10-4.45) times that in the southern-rural regions, while in the northern-urban region it was 2.28 (95% CI: 1.93-2.69) times that in southern-urban region. The PRR of anencephaly was only 1.15 (95% CI: 0.99-1.34) in the northern-urban region as compared to the southern-urban region, but this was not statistically significant.

**Table 2:** Results of Poisson Regression model for estimating the PRR (its 95% CI) between south and north region, and urban and rural areas of China

		**Anencephaly**	**Spina bifid**	**Encephalocel**	**NTDs**
South	Urban	1	1	1	1
	Rural	1.53 (1.30-1.81)	1.40 (1.14-1.73)	1.42 (1.07-1.90)	1.47 (1.31-1.66)
North	Urban	1	1	1	1
	Rural	2.10 (1.81-2.43)	2.29 (2.01-2.60)	1.80 (1.42-2.28)	2.14 (1.96-2.34)
Urban	South	1	1	1	1
	North	1.15 (0.99-1.34)	2.28 (1.93-2.69)	1.39 (1.08-1.77)	1.56 (1.41-1.72)
Rural	South	1	1	1	1
	North	1.58 (1.35-1.84)	3.71 (3.10-4.45)	1.76 (1.34-2.31)	2.26 (2.04-2.52)
Urban		1	1	1	1
Rural		1.82 (1.63-2.04)	2.00 (1.71-2.24)	1.64 (1.36-1.97)	1.87 (1.74-2.01)
South		1	1	1	1
North		1.35 (1.21-1.50)	2.89 (2.56-3.26)	1.54 (1.29-1.85)	1.87 (1.74-2.01)

The adjusted PRR of NTDs between rural and urban areas was 1.47 (95% CI: 1.31-1.66) in the south and 2.14 (95% CI: 1.96-2.34) in the north. The difference in the total PR of anencephaly and spina bifida between rural and urban areas in the north was greater than that in the south (see the Table 2).

## Discussion

Based on the data retrieved from the CBDMN, our study found that in the northern region, especially in the north-rural region, women have a higher risk of NTDs than their counterparts in the southern region. Nevertheless, NTD subtypes have a different geographic distribution at the provincial level.

The results of our study show that geographic disparities exist in total prevalence of NTDs. Northern rural China still has the highest prevalence of NTDs in the world. Similar geographic disparities were also reported in the United Kingdom and the United States [19,20]. Although these studies tried to explain why the geographical differences existed, the true reasons remain unclear. A previous study showed that the mutation frequency of methylene tetrahydrofolate reductase (MTHFR) C677T among people living in northern China was higher than that in southern China and a foetus with this mutated gene had a higher risk of NTDs [21,22]. Nevertheless, this is inadequate to explain the existence of such a significant difference between regions. Approximately 5% of NTD cases have a family history of disease, and therefore, environmental factors and interaction with genetic factors are also the important causes of NTDs. Although the cause of NTDs in humans is still poorly understood,many studies have shown that environmental factors, including maternal infections, vitamin B deficiency, maternal cigarette smoking, maternal exposure to secondhand smoke, alcohol, caffeine, exposure to chemical fertilizers and pesticides, hyperthermia, maternal diabetes, maternal obesity, taking antipyretic drugs or antibiotics, a high consumption of dried or pickled vegetables, and maternal fumonisin exposure, can increase the risk of NTDs [2-4,23-26]. We think the difference in the prevalence of NTDs between the northern and southern regions may be related to the lifestyle of its citizens, hazardous environmental exposure, and the vitamin supplementation from food. For example, in the north, rural areas in particular, coal is the main fuel used for energy processes, which results in substantial indoor air pollution [27]. This is not the case in the south. Additionally, women in the north perform fewer outdoor activities because of cold weather, and therefore, maternal indoor air pollution exposure in the north is more serious than in the south. Folate deficiency, intake of less green vegetables, and wheat and corn-based food in the winter are also important reasons for a high prevalence of NTDs in the north. A previous study showed that among rural women in the northern region, approximately 50% and 43% were deficient for plasma and red blood cell folate, respectively, compared with 6% and 4%, respectively, for rural women in the southern region [28]. Because of the cold climate, residents in the north (especially in the north-rural areas) consume less fresh vegetables and more pickled vegetables than in the south. However, vitamin B deficiency and high consumption of pickled vegetables are two important risk factors for NTDs [4,27]. A higher possibility of consumption of fumonisin-contaminated corn-based food among the northern women may be another reason for the high prevalence in the north [26]. In addition, the report ‘Tobacco Controls in 2007 in China’ shows that the proportion of women in the north who are exposed to secondhand smoke was higher than in the south [29]. This may help explain the disparities in NTD PRs between the northern and southern regions, which are more obvious in the rural areas.

Previous studies simply revealed south–north differences in the total prevalence of NTDs at the regional level. The strength of our study is that it shows, for the first time, that the three subtypes of NTDs have different geographic distributions at the provincial level in China. Not all NTD subtypes are more prevalent in the northern provinces than the southern provinces. The different geographic distributions of NTD subtypes may provide us with some ideas for developing suitable local intervention strategies. It is worthy to note that the most prevalent NTD is anencephaly in the south and spina bifida in the north. The subtype composition of NTDs differs within each country as well. The major NTDs in Iran is anencephaly and spina bifida in the USA, Canada, and New Zealand. [11] It indicates that the causes of the three subtypes of NTD differ and that these differences need further exploration.

An urban–rural disparity in the prevalence of NTDs was also shown in this study. The differences in residents’ education level, health awareness, and maternal nutritional status between urban and rural areas, likely contribute to this disparity. A previous study showed that an educational level of primary school or lower was significantly associated with increased risk for an NTD [27]. It is reported that 90% of illiterate peoples in China reside in the rural areas. A study on folic acid awareness and intake among women in the 6 northern province of China showed that the proportion of women who knew about folic acid and who use folic acid supplements was 73.0% and 10.5%, respectively, in the urban areas, compared with 50.0% and 7.6%, respectively, in the rural areas. [30] A large proportion, 40%, of rural women were deficient for red blood cell folate in the north, compared with 20% in the south. [31] In addition, exposure to chemical fertilisers and pesticides during pregnancy is also associated with a relatively high prevalence of NTDs in rural areas [24].

Our study has some limitations. (1) It is possible that hospital-based samples may introduce a referral bias because approximately 15% of birth cases are delivered in the hospitals at the county level of lower. If the foetus is suspected to have congenital anomalies during the prenatal examination, his or her mother is usually at a higher-level hospital that has the capabilities to diagnosis it. It can be estimated that a proportion of these women are willing to deliver in the higher-level hospital to obtain better medical services. This may lead to an overestimation of the total prevalence of NTDs. However, although cases with congenital anomalies are diagnosed in hospitals at the county level or higher, there is also a proportion of mothers who are willing to deliver in the hospitals at the county level of lower because of their relatively low-cost medical services. These cases were not included in our surveillance system. Additionally, because the data used in this study includes wide geographical coverage and a large sample size, the estimation of the PR of NTDs is relatively stable. (2) The denominator of the prevalence of NTDs was the total number of births (live births and still births at greater than or equal to 28 weeks of GA) in the same area and time period. Although inclusion of induced and spontaneous foetal deaths at less than 28 weeks of GA would more closely approximate the incidence of NTDs, it is very impractical, as these pregnancy outcomes are often inaccurately counted compared to live births and stillbirths. In addition, thenumber of induced and spontaneous foetal deaths are small in comparison to the number of live births and stillbirths and are unlikely to greatly affect prevalence. (3) In our NTDs database, the minimum gestational age of all NTDs cases is 12 weeks. Almost all NTD cases that are spontaneously aborted during the first trimester cannot be detected because of the limits of detection technology in our birth defects surveillance network, and therefore, the total prevalence of NTDs shown in our study is underestimated from this perspective. Besides, the ability to ascertain NTDs is different among the member hospitals at different-levels (county-level, municipal-level hospitals and provincial-level) in CBDMN. Approximately 85% of deliveries in the county-level hospitals came from rural areas and 90% of deliveries in the municipal or provincial level hospitals from urban areas. Relatively low ability to ascertain NTDs in the county-level hospitals may induce to underestimate the total NTDs prevalence in the rural.

## Conclusions

In conclusion, a high total prevalence of NTDs remains one of the major public health concerns in China. Eliminating the geographic and urban–rural disparities in the disease burden of NTDs is a priority for future disease interventions. The high prevalence of NTDs in rural areas (especially in the rural areas of northern China) suggests that more integrated interventions should be implemented in these areas. Campaigns aimed at increasing the awareness, knowledge, and periconceptional use of folic acid, as well as increasing health education about NTD prevention and adequate nutritional intake in pregnancy for women of childbearing age, could all be the effective interventions for NTD prevention. Future studies should focus on community experiments for conducting integrated primary interventions in the high-prevalence areas to develop an effective strategy to eliminate the disparity.

## Competing interests

The authors declare that they have no competing interests.

## Authors’ contributors

XHL and JL made equal contributions, jointly writing the paper. QL, MRL and GXZ participated in data collection, data analysis, and data quality checking and results interpretation. JZ and YPW led the writing, jointly designing the study, and supervising all aspects of its implementation. LD, DZM and HW made critical comments on the drafts. All authors read and approved the final version.

## Authors’ information

Xiaohong Li and Juan Liang joint first authors.

## Pre-publication history

The pre-publication history for this paper can be accessed here:

http://www.biomedcentral.com/1471-2458/13/161/prepub
